# Feasibility and safety of multi-field functional electrical stimulation for distal upper-limb rehabilitation after subacute stroke: a randomised controlled feasibility trial

**DOI:** 10.3389/fresc.2026.1920617

**Published:** 2026-09-11

**Authors:** Silvia Guillén-Climent, Pablo Casado-Adam, Manuela Mejías-Ruiz, Aitor Martín-Odriozola, Cristina Rodríguez-de-Pablo, Inmaculada Salcedo-Leal, Esmeralda Sillero-Ruz, María Dolores Morente-Navajas, Manuel Flores-Lara, José Peña-Amaro, Fernando Jesús Mayordomo-Riera

**Affiliations:** 1 Preventive Medicine and Public Health Group (GA13), Maimónides Biomedical Research Institute of Córdoba (IMIBIC), Reina Sofía University Hospital, University of Córdoba, Córdoba, Spain; 2 Interlevel Clinical Management Unit of Physical Medicine and Rehabilitation, Reina Sofía University Hospital, Guadalquivir Health District, Córdoba, Spain; 3 Clinical Management Unit of Physical Medicine and Rehabilitation, Regional University Hospital of Málaga, Málaga, Spain; 4 University of Málaga, Málaga, Spain; 5 Rehabilitation, Innovation, and Quality of Life (FE-18), Biomedical Research Institute of Málaga (IBIMA), Málaga, Spain; 6 Fesia Technology, Donostia–San Sebastián, Spain; 7 Fesia Clinic, Donostia–San Sebastián, Spain; 8 Department of Physiology, University of the Basque Country (EHU), Leioa, Spain; 9 Preventive Medicine and Public Health Unit, Reina Sofía University Hospital, Córdoba, Spain; 10 Department of Medical and Surgical Sciences, University of Córdoba, Córdoba, Spain; 11 Department of Morphological and Socio-Sanitary Sciences, University of Córdoba, Córdoba, Spain; 12 Maimónides Biomedical Research Institute of Córdoba (IMIBIC), Reina Sofía University Hospital, University of Córdoba, Córdoba, Spain; 13 Department of Applied Physics, Radiology and Physical Medicine, University of Córdoba, Córdoba, Spain

**Keywords:** assistive technology, feasibility study, functional electrical stimulation (FES), hand function, multi-field electrodes, neurorehabilitation, stroke recovery, upper extremity

## Abstract

**Background:**

Functional electrical stimulation (FES) is recommended for upper-limb rehabilitation after stroke but remains underused in routine clinical practice. Multi-field FES may simplify clinical application and improve selective muscle activation, although evidence generated under real-world rehabilitation conditions remains limited.

**Objective:**

To assess the feasibility, usability, and safety of multi-field FES for distal upper-limb rehabilitation after subacute stroke and to generate preliminary effect estimates to inform a future definitive trial.

**Methods:**

We conducted a single-centre, parallel-group, randomised controlled feasibility trial with blinded outcome assessment. Sixteen adults with subacute stroke (≤6 months) and unilateral upper-limb impairment were randomised 1:1 to FES-assisted therapy or conventional rehabilitation (*n* = 8 per group). Both groups received two 40-minute upper-limb therapy sessions per week for 6 weeks (12 sessions); the experimental group received treatment using the Fesia Grasp multi-field stimulation device. Primary outcomes were retention, adherence, usability, motivation, and safety. Secondary exploratory outcomes included the Fugl-Meyer Assessment for Upper Extremity, modified Medical Research Council scale, grip strength, and Box and Block Test, assessed at baseline and post-intervention by blinded evaluators.

**Results:**

Of 20 participants screened between April 2022 and August 2023, 16 were recruited and randomised (8 per group) and received 12 sessions of 40 minutes over 6 weeks. All participants completed the study, resulting in 100% retention and adherence (16/16; 95% CI 79.4–100% for both outcomes). In the FES group, usability and motivation scores were high, indicating good acceptability of the intervention. Two FES participants experienced minor, transient skin erythema after electrode application; no serious adverse events were observed. Exploratory analyses yielded point estimates that favoured the FES group for distal upper-limb motor outcomes; however, confidence intervals were wide and findings should not be interpreted as evidence of clinical efficacy.

**Conclusion:**

Multi-field FES for distal upper-limb rehabilitation after subacute stroke was feasible and well accepted in routine clinical practice, with no serious adverse events observed. These findings provide preliminary data to support the design of future adequately powered efficacy trials.

**Clinical Trial Registration:**

https://clinicaltrials.gov/study/NCT04992910, identifier: NCT04992910.

## 1 Introduction

Stroke is one of the leading causes of long-term disability worldwide (1), and upper-limb impairment affects nearly half of patients in the acute phase and often persists chronically (2). Paresis, abnormal muscle tone, reduced dexterity, and sensory deficits contribute to activity limitations and reduced quality of life, making upper-limb recovery a key priority for patients and clinicians (3).

Functional electrical stimulation (FES) has been used in post-stroke rehabilitation for several decades. Early studies and clinical guidelines suggested potential benefits of FES (4, 5), and subsequent systematic reviews have reported positive effects on motor recovery and activity performance (6, 7). However, despite guideline recommendations and supporting evidence, FES remains underused in routine clinical practice (8, 9). Conventional surface electrode systems have limited selectivity, require time-consuming placement, and typically maintain fixed configurations during therapy sessions. These limitations are particularly relevant in anatomically complex regions such as the forearm, where selective muscle activation is essential for functional grasp and release (9, 10).

To address these challenges, multi-field FES systems have been developed to simplify clinical application and potentially improve functional outcomes through more selective and intuitive muscle activation (10–13). Multi-pad and multi-field electrode arrays were introduced more than a decade ago to enhance selective muscle activation during FES-assisted grasp (14). However, recent technical evidence indicates that achieving fully selective activation of individual forearm muscles remains an important engineering challenge (15). Thus, although multi-field systems may overcome some practical limitations of conventional FES, their clinical feasibility and effectiveness in stroke rehabilitation remain insufficiently established.

The Fesia Grasp device has so far been evaluated in a small pilot study in five patients with subacute stroke (16) and, more recently, in a randomised controlled trial involving patients with multiple sclerosis (17). Separately, one of the largest and most recent multisite randomised trials of FES for upper-limb recovery after stroke evaluated a conventional, non-multi-field electrode configuration in the chronic phase (18). Thus, although contemporary evidence supports continued interest in FES for upper-limb rehabilitation after stroke, evidence on the repeated use of multi-field FES as part of rehabilitation in patients with subacute stroke remains scarce. The present study addresses this gap by evaluating multi-field FES-assisted distal upper-limb rehabilitation in patients with subacute stroke using a parallel-group randomised controlled feasibility design within routine public-hospital rehabilitation.

The aim was to assess feasibility, usability, and safety of the intervention. A secondary exploratory objective was to estimate preliminary effect sizes for distal upper-limb motor outcomes to inform the design of future adequately powered trials.

## 2 Materials and methods

### 2.1 Design

We conducted a parallel-group, randomised controlled feasibility trial with blinded outcome assessors to evaluate Fesia Grasp–assisted hand rehabilitation after stroke. The study followed the CONSORT extension for randomised pilot and feasibility trials (Supplementary Data Sheet 1) (19). Ethical approval was obtained from the Drug Research Ethics Committee of Córdoba, Spain (reference 5041), and the trial was prospectively registered at ClinicalTrials.gov (NCT04992910) before enrolment.

### 2.2 Participants

Participants were recruited from the Physical Medicine and Rehabilitation Unit at Reina Sofía University Hospital (RSUH). Eligible participants were adults (≥18 years) referred for rehabilitation with clinically confirmed ischaemic or haemorrhagic stroke in the subacute phase (≤6 months) and unilateral upper-limb impairment.

Exclusion criteria were: severe proximal upper-limb spasticity (Modified Ashworth Scale >3), psychiatric disorders (including severe depression), cognitive impairment (Mini-Mental State Examination ≤24), comorbidities limiting participation, spatial neglect, cardiac pacemaker, pregnancy, active skin lesions, peripheral neuropathy, clinically significant oedema of the forearm or hand, or pain limiting upper-limb use. All participants provided written informed consent.

**Figure 1:**
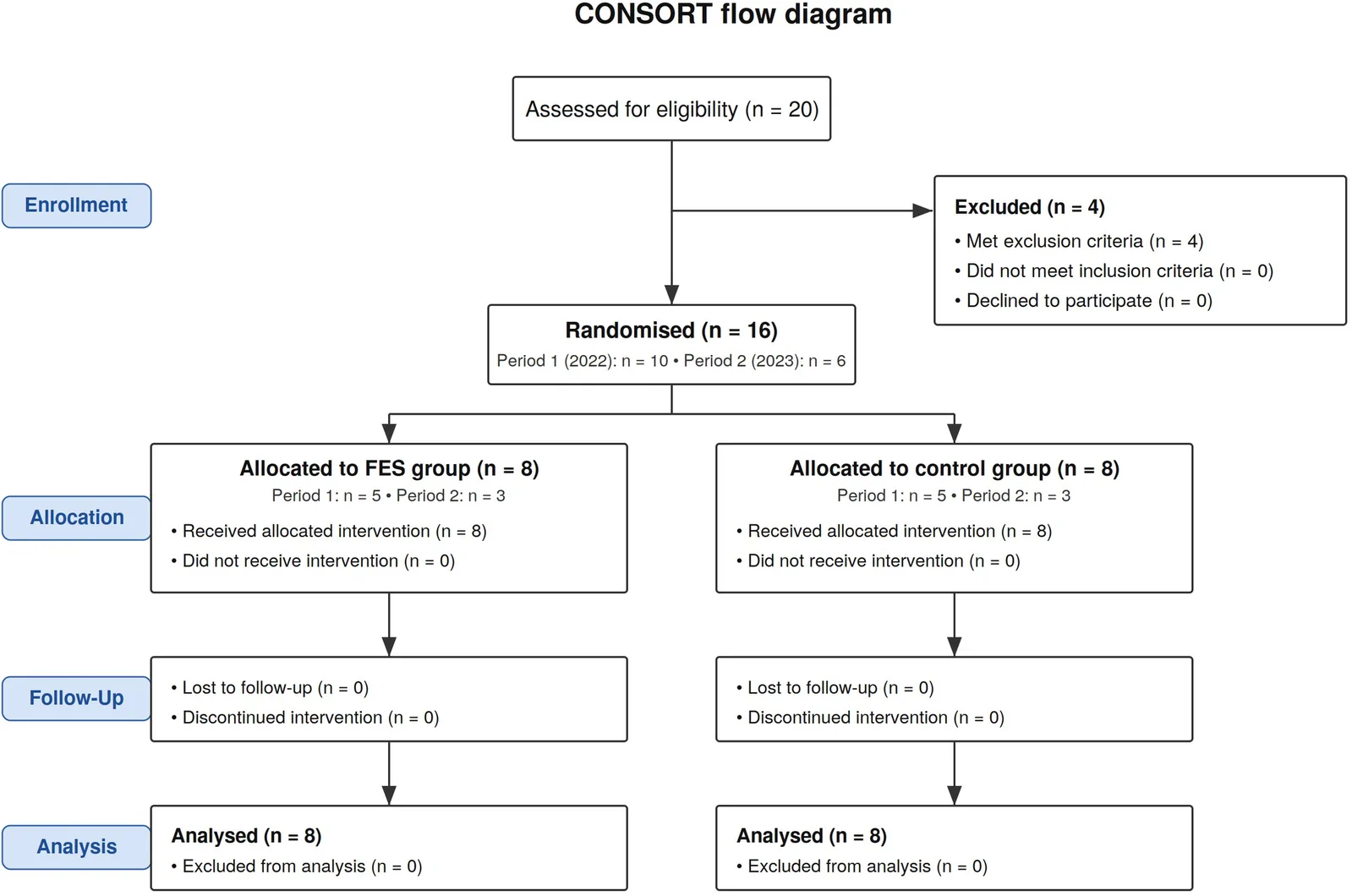
CONSORT 2010 flow diagram.

Participants were randomised to either a control group receiving conventional therapy or an experimental group receiving Fesia Grasp–assisted FES, with equal treatment dose. The participant flow is shown in Figure 1.

### 2.3 Recruitment and randomisation

Participants were recruited consecutively from the Physical Medicine and Rehabilitation Unit at RSUH. Recruitment was conducted in two periods: Period 1 (April-October 2022; *n* = 10) and Period 2 (March-August 2023; *n* = 6), separated by an interruption of approximately 5 months due to the temporary unavailability of a member of the study team. Recruitment was paused during this period and resumed when the same team member returned. Eligibility criteria, interventions, and assessment procedures were identical across periods. The composition of the study team and standard rehabilitation care were unchanged across the two active recruitment periods.

After eligibility confirmation and consent, participants were assigned a sequential code. Randomisation was implemented separately within successive recruitment batches (4, 4, and 2 participants in Period 1; 2, 2, and 2 in Period 2). These batch sizes reflected the number of eligible participants available at each allocation step and were not prespecified permuted block sizes. Within each batch, participant codes were entered into Epidat 4.2 and randomly allocated 1:1 to the study groups. Randomisation was performed by an independent physician (I.S.-L.), who had no involvement in participant recruitment, intervention delivery, or outcome assessment. Importantly, randomisation was performed only after all participants within each batch had been recruited, eligibility confirmed, consent obtained, and participant codes assigned. Physiotherapists were responsible for recruitment and intervention delivery, while outcome assessments were performed by physicians specialised in Physical Medicine and Rehabilitation who were blinded to group allocation.

Participants and therapists were not blinded due to the nature of the intervention. Outcome assessors and data analysts remained blinded throughout and were not involved in other study procedures.

Personnel from Fesia Technology had no contact with participants and no role in the supervision or delivery of therapy sessions; their involvement was strictly limited to providing the device and technical/software support to the clinical team. In addition, blinded outcome assessors had no contact with the device manufacturer at any point during the assessment process.

### 2.4 Sample size

As a feasibility trial, no formal sample size calculation was performed, in accordance with CONSORT guidance for pilot and feasibility studies (19). A pragmatic sample of 16 participants was selected based on the limited availability of eligible patients and the need to obtain preliminary feasibility estimates. Usability considerations followed Nielsen's recommendations for small-sample testing (20).

### 2.5 Functional electrical stimulation system

**Figure 2:**
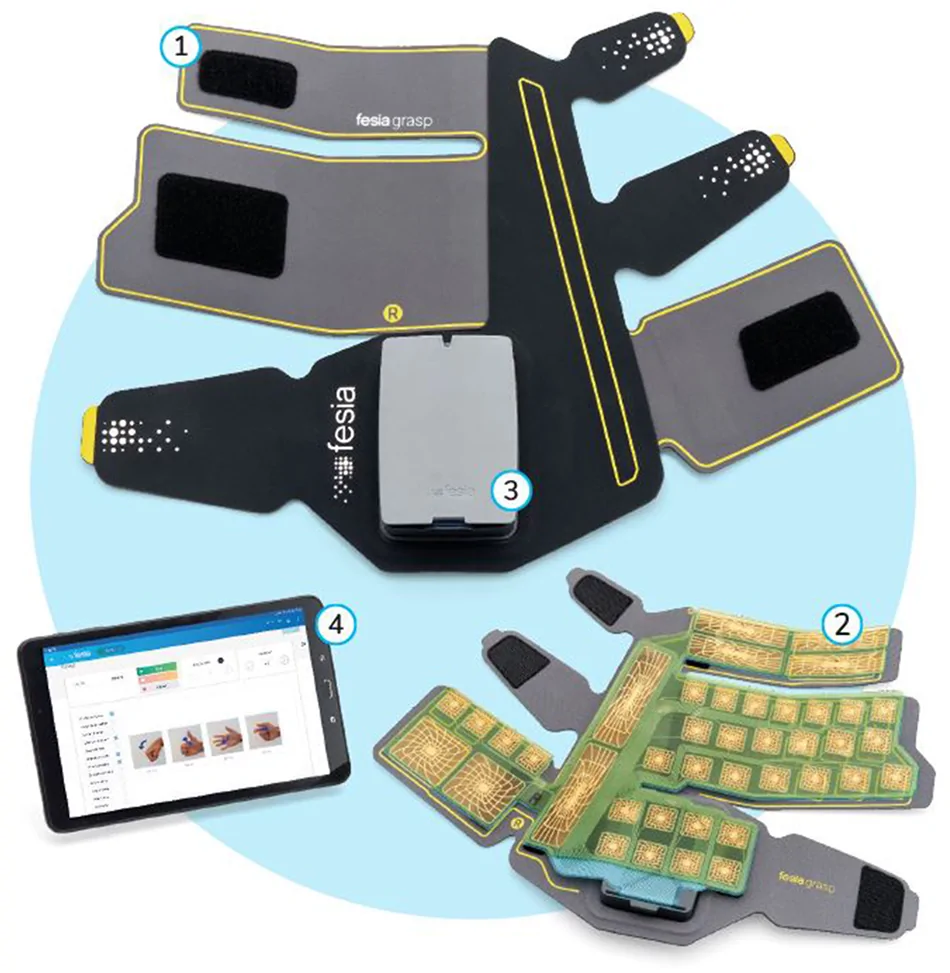
Components of the Fesia Grasp device. 1, textile band; 2, multi-field electrode; 3, stimulator; 4, Fesia Pro software. Image provided by Fesia Technology.

FES was delivered using the Fesia Grasp device (Fesia Technology, Donostia–San Sebastián, Spain), a CE-marked upper-limb neurorehabilitation system (Figure 2). The system includes a portable stimulator, a multi-field forearm electrode, and tablet-based control software (Fesia Pro). The electrode comprises 32 active and 8 dispersive fields, enabling selective forearm muscle activation to produce functional wrist and finger movements (21) (Supplementary Data Sheet 2). Stimulation parameters (current, frequency, pulse width) were individually adjusted within the device range (0–60 mA, 0–40 Hz, 150–300 µs) to elicit targeted movements (16, 21). Parameter selection was tailored to each participant according to the stimulation intensity required to elicit the intended movement while maintaining patient comfort and tolerability. Physiotherapists delivering the intervention received device-specific training from the manufacturer before intervention delivery commenced.

### 2.6 Intervention

Both groups received two 40-minute upper-limb therapy sessions per week for 6 weeks (12 sessions), consistent with standard care at the RSUH Physical Medicine and Rehabilitation Unit. Sessions were delivered individually by physiotherapists in the routine physiotherapy facilities of the Rehabilitation Unit.

#### 2.6.1 Control group

Participants received individual 40-minute conventional upper-limb rehabilitation sessions. Treatment followed institutional protocols and included: (1) neuromuscular preparation (range-of-motion and tone-management exercises), (2) motor control and strengthening of distal upper-limb muscles, and (3) task-oriented training and activities of daily living (ADL) practice using functional object-manipulation tasks. Tone-management techniques were applied when clinically indicated.

#### 2.6.2 FES group

Participants received individual 40-minute upper-limb rehabilitation sessions assisted throughout by Fesia Grasp, which was fitted at the beginning of each session and remained in place until session completion. Stimulation was applied during the therapeutic exercises, with rest intervals between tasks. Sessions followed the same overall therapeutic structure and included: (1) familiarisation and motor recruitment, (2) repetitive task practice with active participation, (3) ADL-oriented functional training, and (4) tone-management when required. Fesia Grasp-assisted practice substituted for the corresponding conventional distal upper-limb treatment within the same 40-minute therapy session; FES was not provided as additional treatment, and total therapy duration and therapist contact time were equivalent between groups.

Stimulation settings and session progression were individualised according to the participant's motor response, functional ability, and tolerance, while maintaining the scheduled 40-minute session duration. No modifications to the intervention protocol were made during the study.

Intervention delivery was monitored through attendance and session-completion records. A full TIDieR (Template for Intervention Description and Replication)-compliant description of the intervention is provided in Supplementary Data Sheet 3.

### 2.7 Assessment

Data were collected and managed using REDCap hosted at FIBICO (Spain), in compliance with the General Data Protection Regulation (22).

#### 2.7.1 Primary outcomes

Feasibility was assessed through retention (≥80% completion of 12 sessions, including the post-intervention assessment) and adherence (≥80% attendance of scheduled sessions). Usability and motivation were assessed post-intervention using a structured usability questionnaire (Supplementary Data Sheet 4) and an adapted, shortened Intrinsic Motivation Inventory (IMI) (23, 24) (Supplementary Data Sheet 5). The usability questionnaire evaluated comfort, ease of use, satisfaction, willingness to continue use, and recommendation to other patients (5-point Likert scale). The IMI assessed Value/Usefulness, Perceived Competence, Interest/Enjoyment, Perceived Choice, Effort/Importance, and Pressure/Tension (1–7 scale). Pressure/Tension was reverse-scored so that higher values reflected lower perceived pressure/tension (23). Adverse events and serious adverse events were recorded at each session and reported according to institutional procedures.

#### 2.7.2 Secondary outcomes

Exploratory efficacy outcomes were assessed at baseline (T0) and post-intervention (T1) by blinded physicians. The principal exploratory motor outcome was the Fugl-Meyer Assessment for Upper Extremity (FMA-UE) (25). Secondary measures included handgrip strength (dynamometry), the modified Medical Research Council scale (mMRC) (26), and the Box and Block Test (BBT) (27).

### 2.8 Statistical analysis

Analyses were performed using R version 4.3.2. Descriptive statistics were used to summarise feasibility, usability, motivation, and safety outcomes. Ordinal variables (mMRC) are presented as median (first to third quartile, Q1–Q3), and continuous variables as mean and standard deviation.

For exploratory clinical outcomes, between-group differences at post-intervention were estimated using analysis of covariance (ANCOVA), with treatment group as the main factor and the corresponding baseline value as a covariate (28). Adjusted mean differences with 95% confidence intervals were reported. As a *post hoc* sensitivity analysis, the ANCOVA models were repeated with recruitment period (first vs. second) included as an additional categorical covariate to assess whether the estimated between-group effects were materially influenced by the two recruitment periods. Given the small number of participants within each period, treatment-by-period interactions were not estimated. Results of these sensitivity analyses are reported in Supplementary Data Sheet 6.

As the modified Medical Research Council (mMRC) scale is ordinal, mMRC outcomes were treated as approximately continuous for baseline-adjusted exploratory estimation within the ANCOVA framework. This approach allowed estimation on a scale comparable with the other clinical outcomes, while recognising that it does not fully account for the ordinal nature of the measure. Given the small sample size and feasibility design, no additional ordinal or non-parametric sensitivity analysis was performed for the mMRC outcomes. Accordingly, these estimates should be considered preliminary and hypothesis-generating, and future adequately powered trials should use methods that explicitly account for the ordered nature of the scale. Adjusted mean differences, 95% confidence intervals, and Hedges’ g (29) were calculated to provide preliminary estimates for future trial planning. Effect sizes were interpreted following Cohen's benchmarks (small ≥ 0.20, moderate ≥ 0.50, large ≥ 0.80) (30). No adjustment for multiple comparisons was applied because all clinical outcome analyses were exploratory. Model assumptions were not formally tested given the limited sample size.

## 3 Results

### 3.1 Participants

Between April 2022 and August 2023, 16 participants were randomised: 10 in the first recruitment period (5 per group) and 6 in the second (3 per group).

Baseline demographic and clinical characteristics by group are summarised in Table 1. Mean age was 71.1 (SD 14.3) years in the control group and 62.8 (SD 8.8) years in the FES group. Time since stroke was similar between groups (3.3 [SD 2.0] vs 3.5 [SD 1.2] months, respectively). Stroke subtype distribution was comparable, with one participant with haemorrhagic stroke in each group. Given the small sample size, some baseline imbalances were present, particularly in age; the greater variability observed in the control group was influenced by one participant aged 90 years.

**Table 1:** Baseline demographic and clinical characteristics by study group.

Characteristic	Control group (*n* = 8)	FES group (*n* = 8)
Age, years, mean (SD)	71.1 (14.3)	62.8 (8.8)
Sex, male/female	3/5	5/3
Hemiparesis side, right/left	3/5	4/4
Time since stroke, months, mean (SD)	3.3 (2.0)	3.5 (1.2)
Stroke subtype, ischaemic/haemorrhagic	7/1	7/1
Baseline FMA-UE total motor score (0–66), mean (SD)	22.0 (12.4)	19.5 (14.7)
Modified Ashworth Scale (MAS) n by category (0/1/1+/2/3)	0/2/1/4/1	1/3/2/1/1

MAS values represent the number of participants in each category, listed in the order 0/1/1+/2/3.

FES, functional electrical stimulation; FMA-UE, Fugl-Meyer Assessment for Upper Extremity; SD, standard deviation.

### 3.2 Primary outcomes: feasibility and usability

Predefined feasibility criteria were met or exceeded, with 100% retention and adherence. All 16 participants completed all 12 scheduled sessions and the post-intervention assessment. In the FES group, all eight participants completed all 12 scheduled FES-assisted sessions, corresponding to 480 minutes of scheduled FES-assisted rehabilitation per participant, with no reported protocol deviations. Usability and motivation were assessed only in the FES group. The results of the structured usability questionnaire are presented in Table 2.

**Table 2:** Structured usability questionnaire outcomes. FES group (*n* = 8).

Domain	Mean (SD)
Comfort of use	4.5 (0.75)
Ease of use	4.75 (0.70)
Satisfaction after use	4.75 (0.46)
Willingness to continue using	5.0 (0)
Recommendation to other patients	5.0 (0)

Ratings based on a 5-point Likert scale (1 = lowest, 5 = highest).

SD, standard deviation.

All usability domains scored 4.5/5 or higher, with ceiling scores (5.0 [SD 0]) for willingness to continue using the device and to recommend it to other patients (Table 2), indicating high acceptability of multi-field FES within routine rehabilitation.

**Figure 3:**
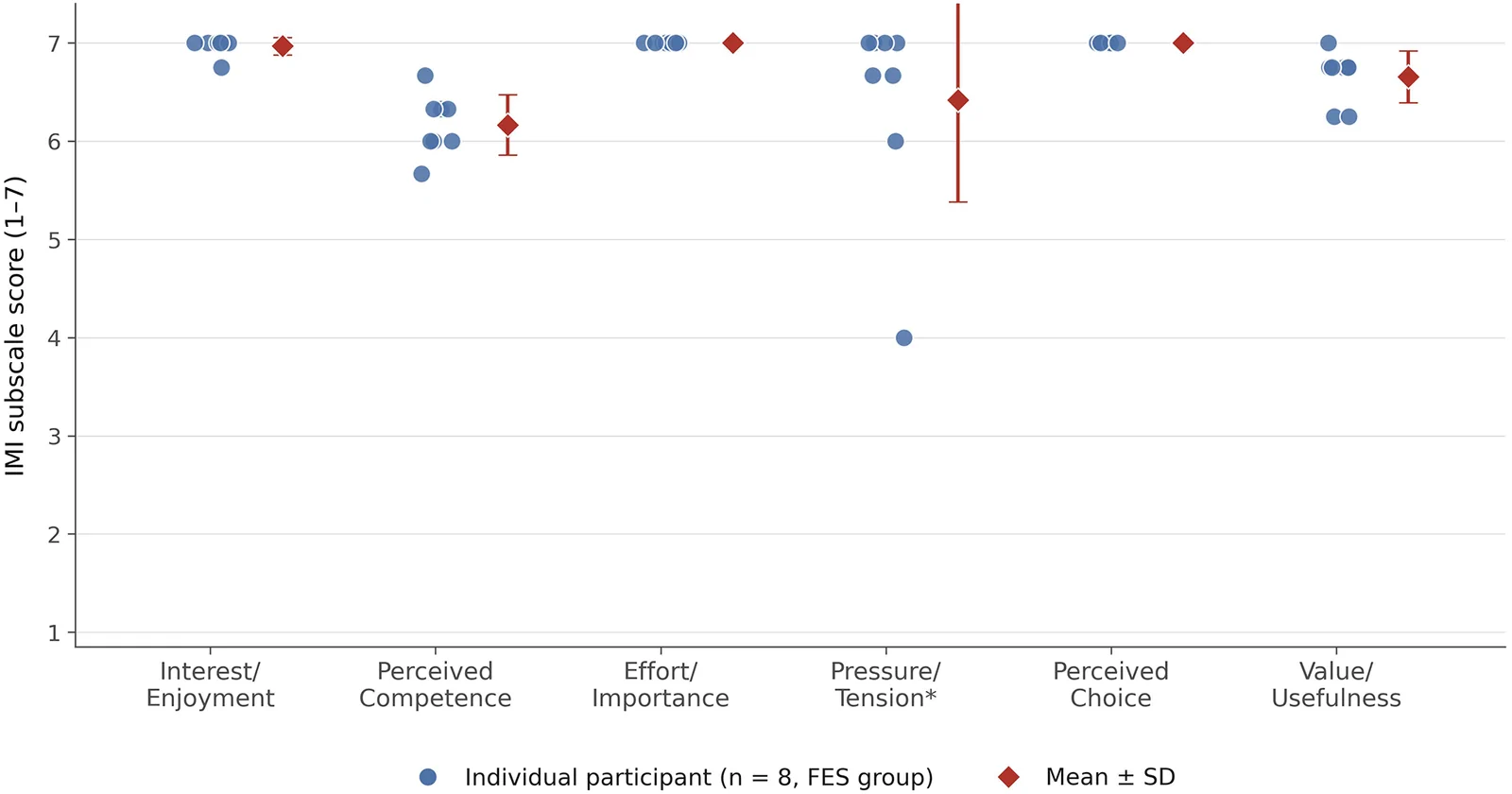
Individual scores (circles) and group mean ± SD (diamonds) for each IMI subscale, FES group (*n* = 8), 1–7 scale (higher = greater motivation). *Pressure/Tension is reverse-scored; higher values indicate lower perceived pressure.

IMI subscale scores were uniformly high (Figure 3): participants rated the intervention as useful (6.66 [SD 0.27]) and themselves as competent (6.17 [SD 0.31]), with low perceived pressure (6.33 [SD 1.02], reverse-scored), high interest and enjoyment (6.97 [SD 0.09]), and ceiling scores for perceived choice and effort/importance (7.0 [SD 0] for both).

Two participants in the FES group experienced minor, transient skin erythema at electrode sites; no serious adverse events occurred.

### 3.3 Secondary outcomes: exploratory clinical outcomes

Detailed results are presented in Table 3. Both groups improved from baseline to post-intervention across most outcomes, consistent with expected recovery during the subacute phase.

**Table 3:** Functional and clinical scores: FMA-UE, mMRC, grip strength, and BBT.

Outcome	Control group (*n* = 8)		FES group (*n* = 8)		Adjusted difference (95% CI)	Hedges’ *g* (95% CI)
	T0	T1	T0	T1		
FMA-UE						
Wrist	2.62 (2.45)	3.00 (2.78)	2.50 (2.27)	5.00 (3.38)	2.15 [0.71, 3.58]	1.52 [0.28, 3.03]
Hand	5.00 (3.82)	6.50 (3.82)	3.88 (3.60)	9.50 (4.28)	3.85 [0.55, 7.16]	1.20 [0.07, 2.27]
Coordination/Speed	1.75 (1.83)	1.88 (2.03)	1.62 (1.60)	1.88 (1.36)	0.10 [−1.05, 1.26]	0.09 [−1.13, 1.25]
Shoulder/elbow/forearm	12.62 (7.84)	13.62 (8.35)	11.50 (9.27)	16.00 (11.01)	3.59 [0.21, 6.98]	1.08 [0.06, 2.10]
Total motor score	22.00 (12.36)	25.00 (13.67)	19.50 (14.73)	32.38 (18.21)	10.15 [3.91, 16.40]	1.66 [0.30, 2.66]
mMRC						
Wrist flexors	3 [0–3.2]	3 [1–3.2]	1.5 [0–3.2]	2.5 [1.8–4.2]	0.81 [0.14, 1.48]	1.23 [0.21, 2.25]
Wrist extensors	3 [0–3]	2.5 [0.8–3.2]	2 [0–3]	3 [1–5]	1.24 [0.16, 2.32]	1.17 [0.15, 2.18]
Finger flexors	3.5 [1.5–4.2]	4 [2.8–4.2]	2 [1–4]	3.5 [3–5]	0.75 [−0.31, 1.81]	0.73 [−0.65, 1.76]
Finger extensors	2 [0–3]	2.5 [0.8–3]	1 [0.8–1.2]	1 [1–3.2]	0.84 [−0.22, 1.89]	0.85 [−0.45, 1.97]
Thumb flexors	2 [0.8–3]	2 [1.8–3.2]	2 [0.8–3.2]	2.5 [2–3.2]	0.16 [−0.72, 1.04]	0.19 [−1.01, 1.28]
Thumb extensors	2.5 [0–3]	2.5 [1.5–3]	0.5 [0–1.5]	2 [0.8–3.2]	0.51 [−0.40, 1.43]	0.58 [−0.86, 1.65]
Grip strength (kg)						
Paretic hand	2.45 (2.05)	3.08 (3.08)	2.66 (2.91)	5.80 (5.52)	2.42 [−0.61, 5.46]	0.81 [−0.21, 1.72]
BBT						
Paretic hand	1.12 (1.89)	2.38 (3.78)	4.00 (7.54)	8.50 (10.23)	2.33 [−0.69, 5.34]	0.82 [−0.63, 1.69]

a) T0: baseline; T1: post-intervention. Values for T0 and T1 are mean (SD), except mMRC values, which are presented as median [first to third quartile, Q1–Q3]. FMA-UE, Fugl-Meyer Assessment for Upper Extremity; mMRC, modified Medical Research Council muscle strength scale; BBT, Box and Block Test; CI: confidence interval.

b) Scoring ranges: FMA-UE total motor score 0–66 (Shoulder/elbow/forearm 0–36, Wrist 0–10, Hand 0–14, Coordination/Speed 0–6); higher scores indicate better motor function. mMRC: 0–5 per muscle group; higher scores indicate greater strength. BBT: number of blocks transferred in 60 s. Grip strength in kilograms.

c) Adjusted differences and Hedges’ g (with small-sample correction) were derived from ANCOVA models (dependent variable: post-intervention score; covariate: baseline score). 95% CIs for both statistics were derived analytically from the ANCOVA residual SD (J = 0.941; df = 13), ensuring full consistency between the two CI columns. Positive values favour the FES group.

d) Estimates are exploratory and not adjusted for multiple comparisons; they should be interpreted cautiously given the limited sample size.

For motor function (FMA-UE), point estimates favoured the FES group across all subscales; the calculated point estimates for Hedges’ g corresponded to large magnitudes on the upper-extremity total score (g = 1.66, 95% CI 0.30-2.66) and on the wrist and hand subscales (g = 1.52 and 1.20), although these exploratory estimates carry wide confidence intervals reflecting the small sample size and should not be over-interpreted as definitive effects (see Table 3). All CIs for Hedges’ g are analytical (see Table 3, note c); at *n* = 8 per group these point estimates carry substantial sampling variability and should be regarded as exploratory only. The point estimate for the proximal (shoulder/elbow/forearm) subscale was also large in magnitude (g = 1.08), whereas coordination and speed showed negligible between-group differences (g = 0.09).

For muscle strength (mMRC), the highest point estimates for effect size were observed at the wrist (flexors g = 1.23; extensors g = 1.17) and at the long finger muscles (extensors g = 0.85; flexors g = 0.73). Effects on thumb musculature were smaller and more uncertain (g = 0.19 to 0.58).

Both functional measures of the paretic hand, grip strength and the BBT, yielded high point estimates for effect size (g = 0.81 and 0.82) coupled with wide and imprecise confidence intervals; variability was substantial, particularly in the FES group, reflecting marked heterogeneity in baseline distal performance.

Sensitivity analyses additionally adjusting for recruitment period yielded between-group estimates that were generally similar to those of the primary baseline-adjusted models. For the FMA-UE total motor score, the adjusted mean difference was 10.15 (95% CI 3.91–16.40) in the primary model and 10.44 (95% CI 5.21–15.67) after adjustment for recruitment period. The largest change was observed for the hand subscale, for which the adjusted mean difference increased from 3.85 (95% CI 0.55–7.16) to 4.48 (95% CI 2.08–6.87). Full sensitivity analyses are presented in Supplementary Data Sheet 6.

## 4 Discussion

This study evaluated a multi-field functional electrical stimulation (FES) system (Fesia Grasp) for upper-limb rehabilitation in subacute stroke. The intervention was feasible within routine clinical practice, with full adherence, no withdrawals, and no serious adverse events. Exploratory between-group analyses provided preliminary estimates of effect on distal upper-limb motor outcomes.

High adherence and absence of dropouts, together with favourable usability and motivation scores, support the feasibility of integrating this intervention into routine rehabilitation. This is particularly relevant given that setup complexity, time constraints, and the limited adoption of rehabilitation technologies more generally are recognised barriers to implementation in clinical practice (8, 9, 31). Comparable feasibility and usability findings have been reported for other flexible surface-FES systems supporting upper-limb task practice after stroke (32). The multi-field electrode design and software-guided parameter selection may reduce setup burden by simplifying electrode placement and adjustment (12). Beyond selective activation, multi-field electrode switching may also reduce operator setup time compared with conventional dual-electrode configurations (10, 33), further supporting the usability advantages observed in this study. Patient motivation is a key determinant of engagement in neurorehabilitation (34), and the high motivation observed may have contributed to the absence of attrition. No serious adverse events were recorded, supporting short-term tolerability within the limits of this small feasibility sample.

Exploratory analyses suggested improvements in distal upper-limb motor outcomes in the FES group. The largest effects were observed for the total FMA-UE score and the wrist and hand subscales, whereas minimal effects were found for the coordination and speed subscale. This may reflect the greater time required for recovery of fine motor control, which often extends beyond the intervention duration (35). A relatively large standardised effect was also observed for the shoulder/elbow/forearm FMA-UE subscale (g = 1.08), despite the intervention primarily targeting distal forearm and hand musculature. In this small subacute sample, improvements across both proximal and distal domains may partly reflect general post-stroke recovery, baseline heterogeneity, or sampling variability. Accordingly, the present findings do not allow a specific mechanistic effect of distal FES to be established.

Substantial within-group variability likely reflects heterogeneity in baseline impairment, typical of small subacute stroke samples. Strength gains were more evident in wrist and finger flexor/extensor muscles than in thumb musculature, possibly due to the greater difficulty of selectively activating intrinsic hand muscles with surface stimulation. Although multi-field electrodes improve selectivity, recruitment of small or deep muscles remains technically challenging (15, 36). Improvements in grip strength and Box and Block Test performance followed a similar pattern to FMA-UE findings, although estimates were imprecise due to sample size and baseline variability.

An imbalance in age between groups should be considered. The control group included one participant aged 90 years, resulting in greater age variability and a higher mean age compared with the FES group. Given the association between age and post-stroke recovery potential, residual confounding cannot be excluded despite statistical adjustment. Notably, because the control group was, on average, older than the FES group, and older age is generally associated with reduced post-stroke recovery potential, this imbalance may have suppressed expected recovery in the control group independent of treatment, potentially inflating rather than attenuating the observed between-group differences favouring the FES group; this residual confounding should be considered when interpreting the exploratory effect estimates. Future trials should consider stratified randomisation by age.

Overall, effect estimates favoured the FES group but should be interpreted as hypothesis-generating. For a definitive trial, sample-size calculation should be based on the observed outcome variability and a prespecified minimal clinically important difference rather than on point estimates from this small feasibility sample.

These findings are consistent with previous evidence supporting FES-based interventions for upper-limb recovery after stroke (4–7, 37–44). However, most prior studies have used conventional electrode configurations with limited selectivity and higher setup demands. Multi-field FES may improve flexibility of muscle recruitment and facilitate task-specific stimulation, although this was not directly tested here.

Observed changes are compatible with proposed mechanisms of FES, including activation of peripheral motor pathways, enhanced somatosensory input, and facilitation of activity-dependent neuroplasticity (45, 46). Increased movement repetition during structured FES sessions compared with conventional therapy may also contribute to recovery (47, 48) and represents a relevant mechanism for future investigation.

Taken together, this study supports the feasibility and acceptability of multi-field FES for distal upper-limb rehabilitation in subacute stroke, with no serious adverse events observed. These findings indicate that multi-field FES appears feasible to implement within routine public-hospital rehabilitation care without major disruption to existing therapy structures, and support the design of a future adequately powered trial to evaluate clinical efficacy.

### 4.1 Limitations

As expected for a feasibility trial, the study was not designed to establish clinical efficacy; therefore, exploratory effect estimates should be interpreted cautiously. The baseline age imbalance and spontaneous recovery during the subacute phase may have influenced the observed changes. Participants and treating physiotherapists could not be blinded, which may have introduced expectancy effects and social-desirability bias in self-reported usability and motivation outcomes. Recruitment occurred in two periods, although adjustment for recruitment period did not materially alter treatment-effect estimates. Exact active stimulation time and session-level parameters were not prospectively recorded. The device-specific usability questionnaire was not formally validated. Finally, the absence of longer-term follow-up and activity-level measures such as the ARAT or WMFT limits assessment of durability and daily-living impact.

### 4.2 Implications for future research

The findings provide feasibility and effect-size data to support the design of future efficacy trials for distal upper-limb rehabilitation after stroke. Future trials should include larger samples, multicentre designs, and longer follow-up periods to confirm efficacy and evaluate durability of effects. Further research should also address stimulation protocol optimisation and the identification of patient subgroups most likely to benefit.

## Data Availability

The raw data supporting the conclusions of this article will be made available by the authors, without undue reservation.

## Supplementary material

Supplementary Data Sheet 1 CONSORT 2010 checklist (feasibility extension).

Supplementary Data Sheet 2 Fesia Grasp device description.

Supplementary Data Sheet 3 TIDieR checklist: detailed description of the intervention.

Supplementary Data Sheet 4 Structured usability questionnaire.

Supplementary Data Sheet 5 Intrinsic Motivation Inventory.

Supplementary Data Sheet 6 Sensitivity analysis adjusting for recruitment period.
