# Supplementary material for: Feasibility and safety of multi-field functional electrical stimulation for distal upper-limb rehabilitation after subacute stroke: a randomised controlled feasibility trial
Source: Front Rehabil Sci. 2026 Sep 11;7:1920617. doi: 10.3389/fresc.2026.1920617 (PMC13613637; doi:10.3389/fresc.2026.1920617)
Supplement: Supplementary file 1 [file Datasheet1.pdf]

Supplementary Material. CONSORT 2010 checklist-extension to randomised pilot and feasibility trials

Manuscript: "Feasibility and safety of multi-field functional electrical stimulation for distal upper-limb rehabilitation after subacute stroke: a randomised controlled trial" (ClinicalTrials.gov NCT04992910).

Locations are reported using manuscript section headings; page numbers have been omitted because pagination may change during production.

Section / Topic	Item	Checklist item	Reported in (section)
**Title and abstract**			
Title and abstract	**1a**	Identification as a pilot or feasibility randomised trial in the title	Title
	**1b**	Structured summary of pilot trial design, methods, results, and conclusions (for specific guidance see CONSORT abstract extension for pilot and feasibility trials)	Abstract
**Introduction**			
Background and objectives	**2a**	Scientific background and explanation of rationale for future definitive trial, and reasons for randomised pilot trial	Introduction
	**2b**	Specific objectives or research questions for pilot trial	Introduction; Abstract
**Methods**			
Trial design	**3a**	Description of pilot trial design (such as parallel, factorial), including allocation ratio	Design; Recruitment and randomisation
	**3b**	Important changes to methods after pilot trial commencement (such as eligibility criteria), with reasons	Design; Recruitment and randomisation
Participants	**4a**	Eligibility criteria for participants	Participants (Methods)
	**4b**	Settings and locations where the data were collected	Participants (Methods); Results
	**4c**	How participants were identified and consented	Participants (Methods); Recruitment and randomisation
Interventions	**5**	The interventions for each group with sufficient details to allow replication, including how and when they were actually administered	Functional electrical stimulation system; Intervention
Outcomes	**6a**	Completely defined prespecified assessments or measurements to address each pilot trial objective specified in 2b, including how and when they were assessed	Assessment; Primary outcomes; Secondary outcomes
	**6b**	Any changes to pilot trial assessments or measurements after the pilot trial commenced, with reasons	Design
	**6c**	If applicable, prespecified criteria used to judge whether, or how, to proceed with future definitive trial	Primary outcomes

Section / Topic	Item	Checklist item	Reported in (section)
Sample size	**7a**	Rationale for numbers in the pilot trial	Sample size
	**7b**	When applicable, explanation of any interim analyses and stopping guidelines	Not applicable
Randomisation: Sequence generation	**8a**	Method used to generate the random allocation sequence	Recruitment and randomisation
	**8b**	Type of randomisation(s); details of any restriction (such as blocking and block size)	Recruitment and randomisation
Allocation concealment mechanism	**9**	Mechanism used to implement the random allocation sequence (such as sequentially numbered containers), describing any steps taken to conceal the sequence until interventions were assigned	Recruitment and randomisation
Implementation	**10**	Who generated the random allocation sequence, who enrolled participants, and who assigned participants to interventions	Recruitment and randomisation
Blinding	**11a**	If done, who was blinded after assignment to interventions (for example, participants, care providers, those assessing outcomes) and how	Recruitment and randomisation
	**11b**	If relevant, description of the similarity of interventions	Not applicable
Statistical methods	**12**	Methods used to address each pilot trial objective whether qualitative or quantitative	Statistical analysis
**Results**			
Participant flow (a diagram is strongly recommended)	**13a**	For each group, the numbers of participants who were approached and/or assessed for eligibility, randomly assigned, received intended treatment, and were assessed for each objective	Participants (Results); Figure 1
	**13b**	For each group, losses and exclusions after randomisation, together with reasons	Participants (Results); Figure 1
Recruitment	**14a**	Dates defining the periods of recruitment and follow-up	Participants (Results)
	**14b**	Why the pilot trial ended or was stopped	Recruitment and randomisation; Sample size
Baseline data	**15**	A table showing baseline demographic and clinical characteristics for each group	Table 1; Participants (Results)
Numbers analysed	**16**	For each objective, number of participants (denominator) included in each analysis. If relevant, these numbers should be by randomised group	Primary outcomes; Secondary outcomes; Tables 2–3
Outcomes and estimation	**17**	For each objective, results including expressions of uncertainty (such as 95% confidence interval) for any estimates. If relevant, these results should be by randomised group	Primary outcomes; Secondary outcomes; Table 3
Ancillary analyses	**18**	Results of any other analyses performed that could be used to inform the future definitive trial	Secondary outcomes; Table 3
Harms	**19**	All important harms or unintended effects in each group (for specific guidance see CONSORT for harms)	Primary outcomes

Section / Topic	Item	Checklist item	Reported in (section)
	**19a**	If relevant, other important unintended consequences	Not applicable
**Discussion**			
Limitations	**20**	Pilot trial limitations, addressing sources of potential bias and remaining uncertainty about feasibility	Limitations
Generalisability	**21**	Generalisability (applicability) of pilot trial methods and findings to future definitive trial and other studies	Limitations; Implications for future research
Interpretation	**22**	Interpretation consistent with pilot trial objectives and findings, balancing potential benefits and harms, and considering other relevant evidence	Discussion
	**22a**	Implications for progression from pilot to future definitive trial, including any proposed amendments	Discussion; Implications for future research
**Other information**			
Registration	**23**	Registration number for pilot trial and name of trial registry	Abstract; Design
Protocol	**24**	Where the pilot trial protocol can be accessed, if available	Design
Funding	**25**	Sources of funding and other support (such as supply of drugs), role of funders	Funding
	**26**	Ethical approval or approval by research review committee, confirmed with reference number	Ethics Statement; Design

Checklist adapted from: Eldridge SM, Chan CL, Campbell MJ, Bond CM, Hopewell S, Thabane L, Lancaster GA; PAFS consensus group. CONSORT 2010 statement: extension to randomised pilot and feasibility trials. BMJ. 2016;355:i5239. doi:10.1136/bmj.i5239. Reproduced and completed under the Creative Commons Attribution (CC BY 3.0) licence.
