# Supplementary material for: Feasibility and safety of multi-field functional electrical stimulation for distal upper-limb rehabilitation after subacute stroke: a randomised controlled feasibility trial
Source: Front Rehabil Sci. 2026 Sep 11;7:1920617. doi: 10.3389/fresc.2026.1920617 (PMC13613637; doi:10.3389/fresc.2026.1920617)
Supplement: Supplementary file 2 [file Datasheet2.pdf]

FESIA GRASP, a functional electrical stimulation system

Fesia Grasp (Fesia Technology, Donostia-San Sebastián, Spain) is a neurological rehabilitation system based on FES, designed to restore upper limb function in patients with neurological conditions, such as stroke, spinal cord injury or multiple sclerosis. This device is the first commercially available FES system for upper limb rehabilitation featuring a multi-field electrode. It delivers trains of asynchronous rectangular biphasic symmetric pulses to different electrode fields in order to generate contractions of the forearm muscles.

The system consists of (1) an ergonomic garment with an integrated (2) multi-field electrode, (3) a portable stimulator, and (4) the Fesia Pro software application (Figure 1). The software application allows configuration of the desired stimulation parameters and monitoring of patient progress.

Figure 1. Fesia Grasp Device

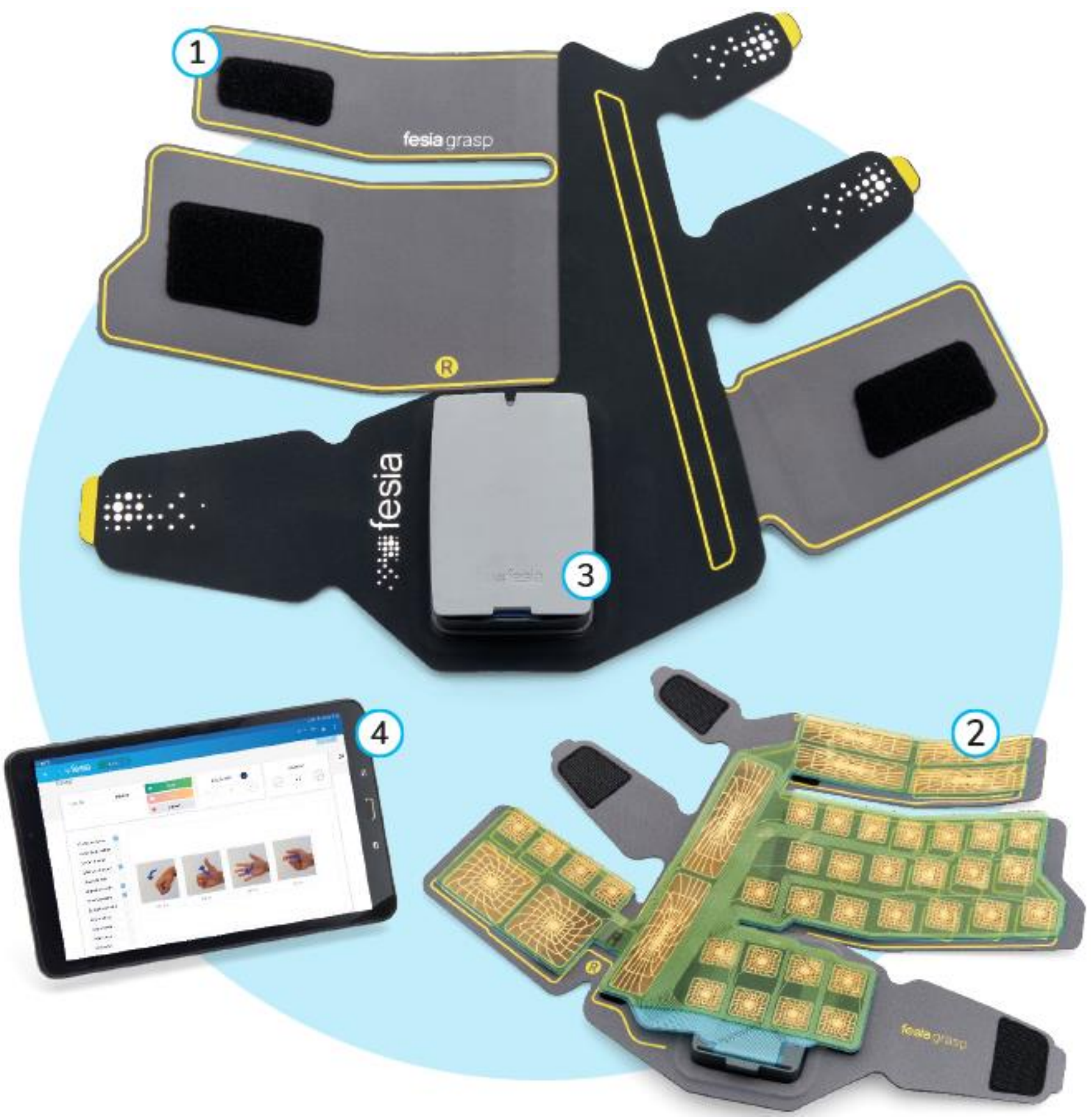

The multi-field electrode includes 32 electrodes and 8 dispersive electrodes, enabling the generation of selective (1 muscle activated) and complex (2 or more muscles activated) hand movements. Despite the relatively high number of electrodes, placement is not time-consuming since the multi-field array is applied in a single step using simple anatomical landmarks, which facilitates its clinical use (Figure 2).

Figure 2. Multi-field electrode placement

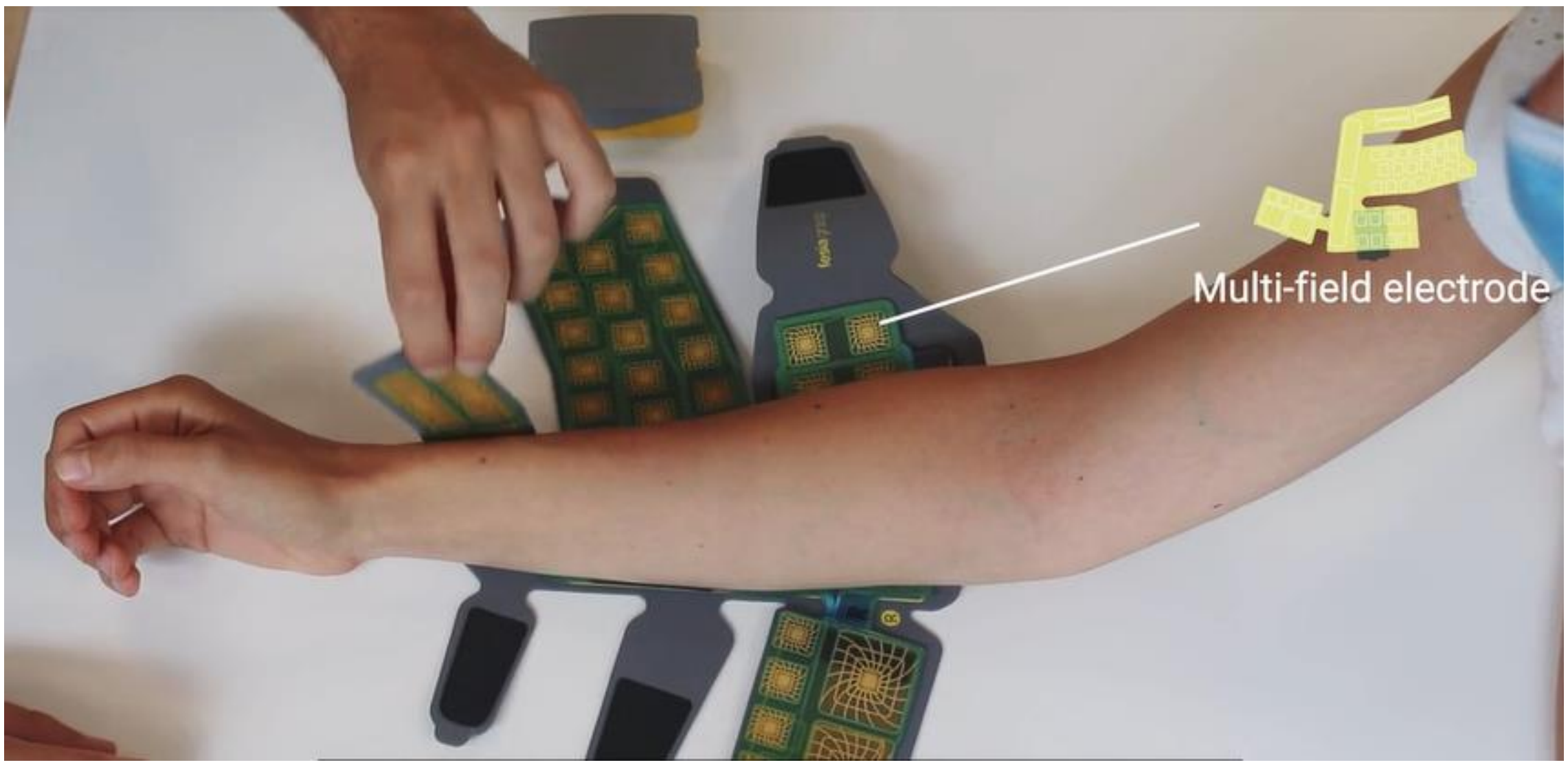

Stimulation Programs

The Fesia Pro software (Figure 3) enables implementation and customization of four standardized stimulation programs, allowing interventions to be tailored to individual patient needs:

1. *Familiarization and Motor Recruitment Program***.** This program provides a progressive introduction to electrical stimulation and allows optimization of stimulation parameters. The electrode fields are systematically scanned while gradually increasing intensity until the motor threshold is reached. Responsive muscles are identified, and individualized stimulation settings are established to ensure reproducible muscle activation in subsequent sessions.

2. *Spasticity Management Program.* This program employs frequency-dependent neuromodulation: low-frequency stimulation (10 Hz) of hypertonic flexors targets fatigue-induced tone reduction through sustained motor unit activation, while higher-frequency stimulation (30 Hz) of antagonist extensors promotes reciprocal inhibition via Ia inhibitory interneurons.
3. *Functional Re-education Program (Repetitive Task Training).* This program focuses on task-specific, repetitive training of the forearm musculature. Stimulation is selectively applied to agonist and antagonist muscles around the same joint, alternating activation to promote controlled and coordinated movement patterns. Stimulation can be combined with voluntary muscle activity to enhance active engagement and motor relearning.
4. *Activities of Daily Living (ADL) Integration Program.* This program aims to transfer motor improvements to meaningful daily activities. It applies real-time adaptive stimulation, dynamically modulated to provide only the minimal assistance required for functional tasks such as grasping and transporting objects. This approach combines functional support with voluntary contribution, promoting autonomy and consolidation of therapeutic gains in everyday performance.

Figure 3. Fesia Pro software interface

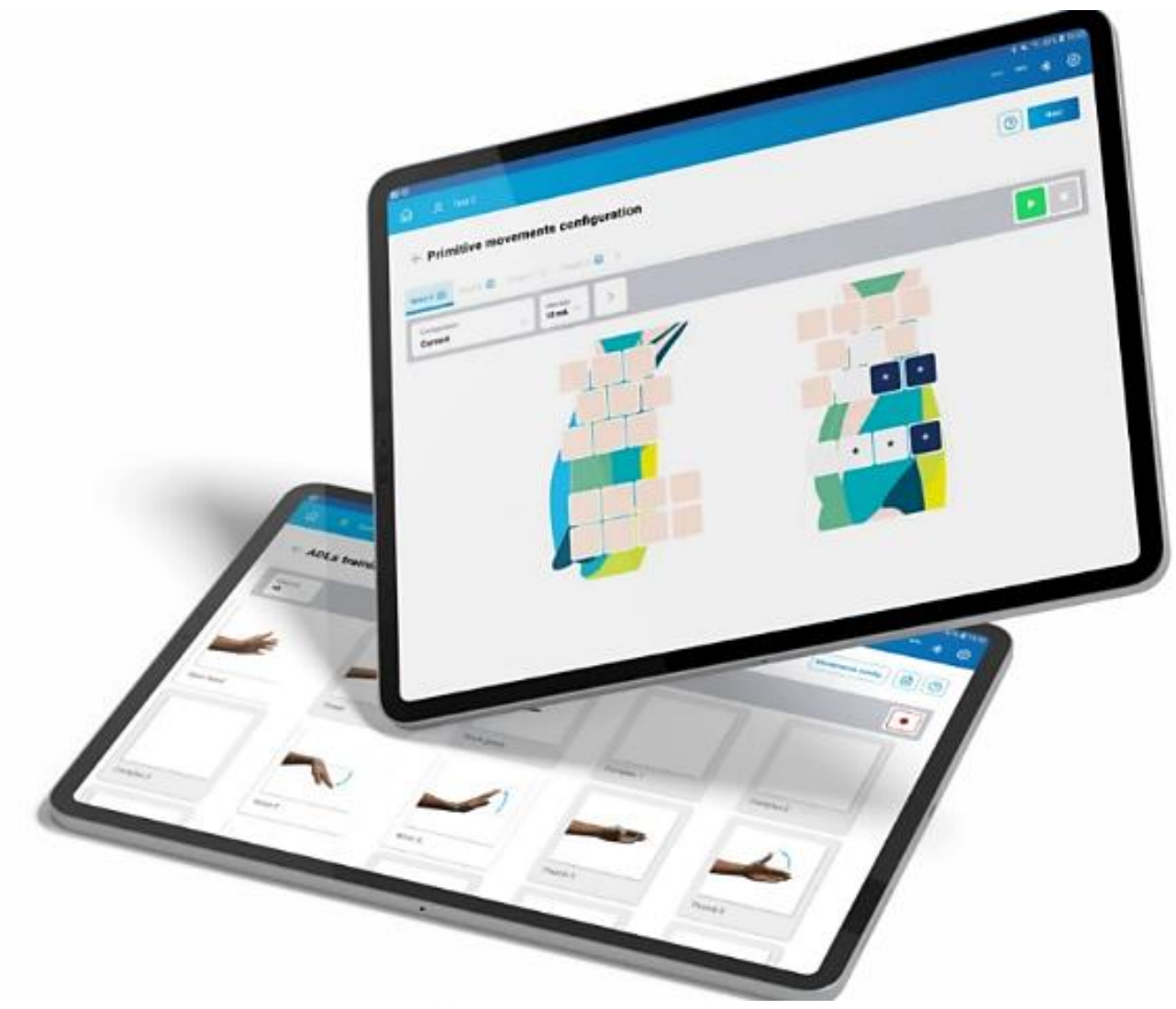

This innovative design offers a very high muscle selectivity (Figure 4), allowing the precise isolation of at least eight selective movements: wrist flexion and extension, thumb flexion and extension, index finger flexion and extension, as well as flexion and extension of fingers 3, 4, and 5. All these movement configurations are performed and managed through the Fesia Pro software described above.

Two or more selective movements can be generated at the same time, creating combinations that allow for the execution of distinct complex movements which are essential for daily activities. These combined movement patterns integrate smaller joint actions, like finger or wrist movements, with specific stimulation timings for each component within the pattern, as well as progressive intensity ramps for both onset and offset, resulting in more physiological activation. Some of the complex movements that can be generated are:

- Hand opening: Simultaneous extension of thumb, index, and remaining fingers.
- Palmar grasp: A combination of thumb, index, and finger extension, followed by flexion, allowing objects to be grasped.
- Pinch grip: A coordinated activation of thumb and index extension and flexion to facilitate pinching movements.

Figure 4. High selectivity of movements

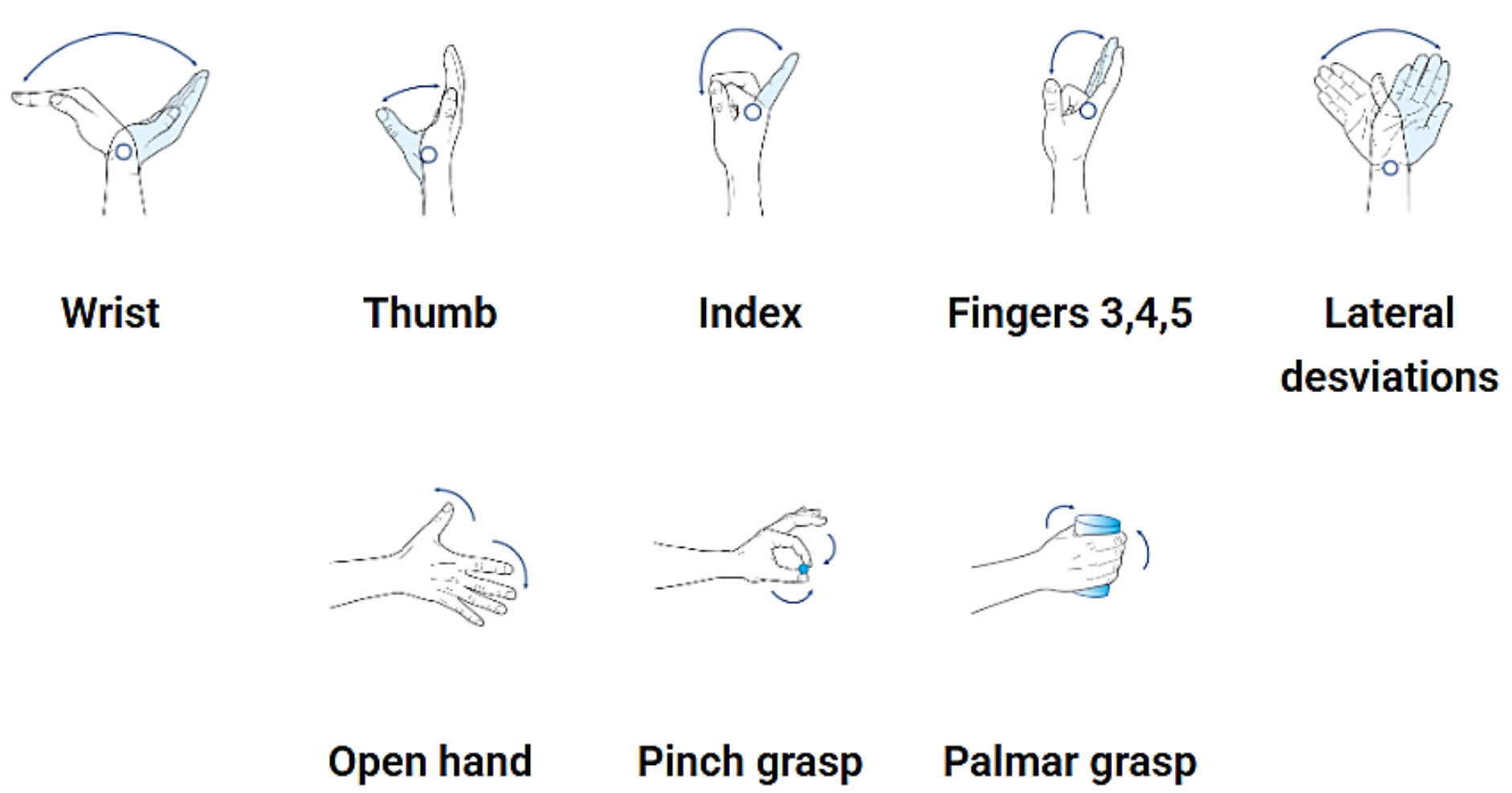

This increased selectivity could enhance the precision and effectiveness of stimulation, enabling a greater variety of movement patterns which are key for functional rehabilitation. Additionally, this system offers adjustable stimulation parameters, with an intensity range of 0 to 60 milliamperes (mA), a frequency range of 0 to 40 Hz, and a pulse width adjustable between 150 and 300 microseconds (µs).

Combining Fesia Grasp with other therapies, such as mirror therapy, significantly enhances grip strength, motor coordination, and functional hand use. In addition, the integration of interactive exercises in the software increases patient motivation and therapy adherence, making rehabilitation a more engaging process.

Beyond their clinical effectiveness and enhanced muscular selectivity, the multi-field electrodes offer significant advantages in terms of usability. These electrodes can also help to reduce muscle fatigue by distributing the stimulation more efficiently, providing a more comfortable sensation for the user. Additionally, they enable the activation of highly repetitive movements, even when the upper limb is positioned differently, making them ideal for intensive rehabilitation.
