# Supplementary material for: Feasibility and safety of multi-field functional electrical stimulation for distal upper-limb rehabilitation after subacute stroke: a randomised controlled feasibility trial
Source: Front Rehabil Sci. 2026 Sep 11;7:1920617. doi: 10.3389/fresc.2026.1920617 (PMC13613637; doi:10.3389/fresc.2026.1920617)
Supplement: Supplementary file 3 [file Datasheet3.pdf]

Supplementary Material. TIDieR checklist for intervention description

TIDieR item	Description in the present study	Where reported
**1. Brief name**	Multi-field FES-assisted upper-limb rehabilitation using the Fesia Grasp system.	Methods 2.5-2.6
**2. Why**	Multi-field FES was used as a rehabilitation aid designed to facilitate activation of forearm musculature and support repetitive, task-oriented practice of wrist, hand, and finger movements during subacute stroke rehabilitation.	Introduction; Methods 2.5
**3. What: materials**	Fesia Grasp system comprising a portable stimulator, a multi-field forearm electrode with 32 active and 8 dispersive fields, and Fesia Pro tablet-based control software.	Methods 2.5; Supplementary Material 2
**4. What: procedures**	Fesia Grasp was fitted at the beginning of each 40-minute experimental session and remained in place until session completion. Stimulation was delivered during therapeutic exercises, with rest intervals between tasks. Sessions comprised familiarisation and motor recruitment, repetitive task practice with active participation, ADL-oriented functional training, and tone management when clinically indicated. Fesia Grasp-assisted practice substituted for the corresponding conventional distal upper-limb treatment within the same 40-minute session; FES was not provided as additional treatment, and total therapy duration and therapist contact time were equivalent between groups.	Methods 2.6
**5. Who provided**	The intervention was delivered by physiotherapists who received device-specific training from the manufacturer before intervention delivery commenced.	Methods 2.5-2.6
**6. How**	Individual, face-to-face physiotherapy sessions.	Methods 2.6
**7. Where**	Routine physiotherapy facilities of the Physical Medicine and Rehabilitation Unit at RSUH.	Methods 2.6
**8. When and how much**	Two scheduled 40-minute upper-limb rehabilitation sessions per week for 6 weeks (12 sessions; 480 minutes of scheduled FES-assisted rehabilitation per participant). Fesia Grasp remained fitted throughout each session, but stimulation was delivered during therapeutic exercises rather than continuously.	Methods 2.6
**9. Tailoring**	Stimulation settings were individually adjusted according to the stimulation required to elicit the intended movement while maintaining participant comfort and tolerability. Session progression was individualised according to motor response, functional ability, and tolerance.	Methods 2.5-2.6
**10. Modifications**	No modifications to the intervention protocol were made during the study.	Methods 2.6
**11. How well: planned**	Intervention delivery was monitored through attendance and session-completion records. Attendance and completion of each scheduled session were documented.	Methods 2.6
**12. How well: actual**	All 8 participants allocated to FES completed all 12 scheduled sessions (96/96 participant-sessions), corresponding to 480 minutes of scheduled FES-assisted rehabilitation per participant. Exact active stimulation time and session-level stimulation parameters were not prospectively recorded.	Results 3.2; Limitations 4.1

Note: This checklist follows the Template for Intervention Description and Replication (TIDieR) framework to support replication of the FES-group intervention. The control-group intervention is described separately in Methods 2.6. FES: functional electrical stimulation; ADL: activities of daily living; RSUH: Reina Sofia University Hospital.
