# Supplementary material for: Feasibility and safety of multi-field functional electrical stimulation for distal upper-limb rehabilitation after subacute stroke: a randomised controlled feasibility trial
Source: Front Rehabil Sci. 2026 Sep 11;7:1920617. doi: 10.3389/fresc.2026.1920617 (PMC13613637; doi:10.3389/fresc.2026.1920617)
Supplement: Supplementary file 4 [file Datasheet4.pdf]

SATISFACTION AND MOTIVATION DURING FESIA GRASP THERAPY

Record ID: ______________________________

Instructions

The following statements express certain thoughts, beliefs, and feelings regarding the exercise you have been performing. Please indicate the degree to which these statements are true for you using the following scale as a guide:

Scale:

- 1 = Strongly Disagree
- 2 = Disagree
- 3 = Neither Agree nor Disagree
- 4 = Agree
- 5 = Strongly Agree

Items

Item	Statement	Rating
1	The FESia Grasp system is comfortable to use	1 2 3 4 5
2	The FESia Grasp system is easy to use	1 2 3 4 5
3	I am satisfied after using the FESia Grasp system	1 2 3 4 5
4	I would be willing to continue using this system for a longer period	1 2 3 4 5
5	I would recommend its use to other patients	1 2 3 4 5

Note: FESia Grasp refers to Functional Electrical Stimulation (FES) therapy system for grasp rehabilitation.
