# Supplementary material for: Feasibility and safety of multi-field functional electrical stimulation for distal upper-limb rehabilitation after subacute stroke: a randomised controlled feasibility trial
Source: Front Rehabil Sci. 2026 Sep 11;7:1920617. doi: 10.3389/fresc.2026.1920617 (PMC13613637; doi:10.3389/fresc.2026.1920617)
Supplement: Supplementary file 5 [file Datasheet5.pdf]

ADAPTED INTRINSIC MOTIVATION INVENTORY

For each of the following statements, please indicate how true it is for you, using the following scale:

1	2	3	4	5	6	7
Not at all true			Somewhat true			Very true

**Questions**	**1**	**2**	**3**	**4**	**5**	**6**	**7**
1. I enjoyed doing this activity very much.							
2. I think I am pretty good at this activity.							
3. I put a lot of effort into this.							
4. I did not feel nervous at all while doing this.							
5. I thought this was a boring activity. (R)							
6. I believe this activity could be of some value to me.							
7. I believe I had some choice about doing this activity.							
8. I didn't try very hard to do well at this activity. (R)							
9. I think that doing this activity is useful for *my upper limb*							
10. This activity did not hold my attention at all.(R)							
11. I felt very tense while doing this activity.							
12. I felt like it was not my own choice to do this task. (R)							
13. I thought this activity was quite enjoyable							
14. I would be willing to do this again because it has some value to me.							
15. I am satisfied with my performance at this task.							
16. It was important to me to do well at this task.							
17. I did this activity because I wanted to.							
18. This was an activity that I couldn’t do very well. (R)							
19. I felt pressured while doing these.							
20. I think doing this activity could help me *to recover agility in my movements.*							
