# Supplementary material for: Feasibility and safety of multi-field functional electrical stimulation for distal upper-limb rehabilitation after subacute stroke: a randomised controlled feasibility trial
Source: Front Rehabil Sci. 2026 Sep 11;7:1920617. doi: 10.3389/fresc.2026.1920617 (PMC13613637; doi:10.3389/fresc.2026.1920617)
Supplement: Supplementary file 6 [file Datasheet6.pdf]

Supplementary Material. Sensitivity analysis adjusting for recruitment period

As a post hoc sensitivity analysis, the main ANCOVA models were repeated with recruitment period (Period 1 vs Period 2) included as an additional categorical covariate. Treatment-by-period interactions were not estimated because of the small number of participants within each recruitment period. This analysis was intended to examine whether adjustment for recruitment period materially altered the estimated treatment effects rather than to formally test between-period differences.

Outcome	Primary ANCOVA: treatment effect, adjusted difference (95% CI)	Sensitivity ANCOVA adjusted for recruitment period: treatment effect, adjusted difference (95% CI)	Recruitment-period coefficient Period 2 vs Period 1 (95% CI)
**FMA-UE**			
Shoulder/elbow/forearm	3.59 [0.21, 6.98]	3.61 [0.14, 7.08]	1.29 [−2.37, 4.95]
Wrist	2.15 [0.71, 3.58]	2.17 [0.77, 3.57]	1.24 [−0.70, 3.17]
Hand	3.85 [0.55, 7.16]	4.48 [2.08, 6.87]	5.71 [2.37, 9.05]*
Coordination/speed	0.10 [−1.05, 1.26]	0.10 [−1.07, 1.28]	−0.48 [−1.71, 0.75]
Total motor score	10.15 [3.91, 16.40]	10.44 [5.21, 15.67]	7.14 [1.22, 13.06]*
**mMRC**			
Wrist flexors	0.81 [0.14, 1.48]	0.82 [0.12, 1.52]	0.15 [−0.81, 1.12]
Wrist extensors	1.24 [0.16, 2.32]	1.40 [0.43, 2.37]	1.59 [0.003, 3.18]*
Finger flexors	0.75 [−0.31, 1.81]	0.73 [−0.41, 1.87]	−0.12 [−1.89, 1.65]
Finger extensors	0.84 [−0.22, 1.89]	0.83 [−0.31, 1.96]	−0.06 [−1.40, 1.28]
Thumb flexors	0.16 [−0.72, 1.04]	0.18 [−0.73, 1.09]	−0.51 [−2.25, 1.23]
Thumb extensors	0.51 [−0.40, 1.43]	0.46 [−0.49, 1.42]	−0.41 [−1.69, 0.87]
**Grip strength**			
Paretic hand (kg)	2.42 [−0.61, 5.46]	2.44 [−0.57, 5.44]	−1.72 [−4.87, 1.42]
**BBT**			
Paretic hand	2.33 [−0.69, 5.34]	2.39 [−0.74, 5.51]	−0.91 [−4.10, 2.27]

** The 95% CI for the recruitment-period coefficient excludes zero. These post hoc coefficients were not prespecified for inferential testing and should be interpreted cautiously.*

Across the 13 outcomes, treatment-effect estimates were generally similar after adjustment for recruitment period. Adjustment for period therefore did not materially alter the estimated treatment effects. Given the small sample size and the post hoc nature of this analysis, residual cohort or period effects cannot be excluded.

Note: Main ANCOVA models included treatment group and the corresponding baseline score. Sensitivity models additionally included recruitment period. Positive treatment-effect estimates favour the FES group. The recruitment-period coefficient represents Period 2 relative to Period 1 and should be interpreted descriptively; no treatment-by-period interaction was estimated. All estimates are exploratory and no adjustment for multiple comparisons was performed. FMA-UE, Fugl-Meyer Assessment for Upper Extremity; mMRC, modified Medical Research Council scale; BBT, Box and Block Test; CI, confidence interval.
